# Assessment of Culturable Tea Rhizobacteria Isolated from Tea Estates of Assam, India for Growth Promotion in Commercial Tea Cultivars

**DOI:** 10.3389/fmicb.2015.01252

**Published:** 2015-11-10

**Authors:** Jintu Dutta, Pratap J. Handique, Debajit Thakur

**Affiliations:** ^1^Microbial Biotechnology Laboratory, Life Sciences Division, Institute of Advanced Study in Science and TechnologyGuwahati, India; ^2^Department of Biotechnology, Gauhati UniversityGuwahati, India

**Keywords:** tea rhizosphere, plant growth promoting rhizobacteria, PGP traits, 16S rRNA, tea growth promotion

## Abstract

In the present study, 217 rhizobacterial isolates were obtained from six different tea estates of Assam, India and subjected to preliminary *in vitro* plant growth promotion (PGP) screening for indole acetic acid (IAA) production, phosphate solubilization, siderophore production and ammonia production. Fifty isolates showed all the PGP traits and five isolates did not exhibit any PGP traits. These 50 potential isolates were further analyzed for quantitative estimation of the PGP traits along with the aminocyclopropane-1-carboxylate (ACC) deaminase, protease and cellulose production. After several rounds of screening, four rhizobacteria were selected based on their maximum ability to produce *in vitro* PGP traits and their partial 16S rRNA gene sequence analysis revealed that they belong to *Enterobacter lignolyticus* strain TG1, *Burkholderia* sp. stain TT6, *Bacillus pseudomycoides* strain SN29 and *Pseudomonas aeruginosa* strain KH45. To evaluate the efficacy of these four rhizobacteria as plant growth promoters, three different commercially important tea clones TV1, TV19, and TV20 plants were inoculated with these rhizobacteria in greenhouse condition and compared to the uninoculated control plants. Though, all the rhizobacterial treatments showed an increase in plant growth compared to control but the multivariate PCA analysis confirmed more growth promotion by TG1 and SN29 strains than the other treatments in all three clones. To validate this result, the fold change analysis was performed and it revealed that the tea clone TV19 plants inoculated with the *E. lignolyticus* strain TG1 showed maximum root biomass production with an increase in 4.3-fold, shoot biomass with increase in 3.1-fold, root length by 2.2-fold and shoot length by 1.6-fold. Moreover, two way ANOVA analysis also revealed that rhizobacterial treatment in different tea clones showed the significant increase (*P* < 0.05) in growth promotion compared to the control. Thus, this study indicates that the potential of these indigenous plant growth promoting rhizobacteria isolates to use as microbial inoculation or biofertilizer for growth promotion of tea crops.

## Introduction

The rhizosphere is the narrow dynamic zone of soil influenced by plant roots where intense plant-microbe interaction is found. These microorganisms can have beneficial effects on the plant health like plant growth and nutrition in agro-ecosystems (Philippot et al., 2013). There are different distinct microbial communities present in the rhizosphere and one of them is well known as plant growth promoting rhizobacteria (PGPR). The PGPR is a group of beneficial soil bacteria associated with the plant roots which can promote plant growth both directly and indirectly (Glick, 1995). The PGPR may influence the plant growth directly by nitrogen fixation, different phytohormones production, phosphate solubilisation and sequester iron by siderophore production while indirectly stimulate the plant growth by producing antifungal metabolites by preventing different phytopathogens (Glick and Bashan, 1997). Therefore, PGPR are very important in improving plant growth and development or abiding in multiple biotic and abiotic stresses, hence the use of PGPR can help in developing eco-friendly practices for sustainable agriculture. A diverse range of bacterial genera such as *Arthrobacter, Azospirillum*, *Azotobacter*, *Bacillus*, *Pseudomonas*, *Klebsiella*, *Burkholderia*, *Erwinia*, *Flavobacterium*, *Micrococcous*, *Enterobacter*, *Xanthomonas*, *Chromobacterium*, *Serratia*, and *Caulobacter* have been documented to promote plant growth (Bhattacharyya and Jha, 2012; Bal et al., 2013).

In the recent years, PGPR got more attention and it has been used as potent biofertilizers (Richardson et al., 2009; Compant et al., 2010). The extensive use of chemical fertilizers has harmful effect on soil health by destabilizing soil fertility and beneficial microbial population (Kalia and Gosal, 2011). In Asia, most of the agricultural fields are highly fertilized with enormous quantities of chemical fertilizers and herbicides for enhancing crop production. Despite its efficiency, the long-term applications of such fertilizers have proved to be perilous to soil health as well as the human and also reduced the crops quality (Islam et al., 2013). Therefore, alternative biotechnological approaches are adapted in different agriculture practices to not only increase the crop production and plant growth, but also to maintain soil health (Fernando et al., 2005).

Though PGPR has been reported previously on different agricultural crops like rice (Sudha et al., 1999), tomato (Mena-Violante and Olalde-Portugal, 2007), wheat (de Freitas, 2000), maize (Biari et al., 2008), canola (Naderifar and Daneshian, 2012) but research of PGPR on tea plants is still poorly explored especially in the Northeast region of India. Tea plant [*Camellia sinensis* (L.) O. Kuntze] of family Theaceae plantation is one of the oldest organized practices in India with massive plantation in the Northeast corner of the agroclimatic belt. Assam is the largest tea producer state in India and it is divided into two main parts; Brahmaputra Valley and Barak Valley (Arya, 2013). The productivity of tea is decreased due to intensive application of chemical fertilizers for a prolonged period (Chakraborty et al., 2013). Hence there is a need to explore the indigenous microflora associated with the tea rhizosphere soil to not only reduce the use of chemical fertilizer but also for the benefit of plant and soil health.

The present study was designed to evaluate the potential of tea root associated bacteria isolated from six different tea estates of Assam, India for plant growth promotion (PGP). The most efficient rhizobacterial isolates were selected on the basis of their *in vitro* PGP experiments and characterized by 16S rRNA gene sequencing. The efficacy of these selected rhizobacterial isolates to use as potential biofertilizer was further evaluated by greenhouse experiment using three Tocklai vegetative (TV) tea clones TV1, TV19, and TV20 which are most extensively cultivated for commercial tea production in the tea estates of Northeast India. During the past years starting from 1949, 31 TV series tea clones and 153 location specific garden series clones have been released by Tocklai Tea Research Institute (TTRI), Tea Research Association (TRA), Jorhat, Assam, India to the tea industry for commercial cultivation. In Northeast India, 60% of the total tea growing areas are covered by planting materials released by TTRI (Das et al., 2012). TV1, TV19, and TV20 are the most popular TV series tea clones grown by the tea estates in Northeast India in terms of productivity and quality of made tea.

## Materials and Methods

### Sample Collection and Isolation of Rhizobacteria

Tea rhizosphere soil samples were collected from six different tea estates of Assam, India, i.e., Sonapur tea estate (26°06′56.40″N91°58′33.18″E), Khetri tea estate (26°06′53.81″N 92°05′27.74″E), Tocklai tea growing area (26°45′18.40″N94°13′16.92″E), Difaloo tea estate (26°36′29.41″N 93°35′03.96″E), Teok Tata tea estate (26°36′29.41″N 94°25′42.59″E) and Hathikuli tea estate (26°34′55.94″N 93°24′43.15″E) during January to April, 2013. The soil samples were collected from 5 to 30 cm depth along with the tea roots. To collect soil samples total area of each tea estate was divided into five blocks. In each block, samples were collected randomly from selected five adult tea plants containing roots and roots adhered soil. The samples collected from each block were mixed to make one composite sample resulting in a total of five composite samples. Soil samples were carried to the laboratory in sterile plastic bags stored at 4°C to process for bacterial isolation. Bacterial isolation of collected samples was completed within 5 days of collection.

For rhizobacterial isolation, 1 g of the soil with roots were suspended in 100 ml of saline solution (NaCl 9 gl^-1^) and then kept in shaking condition (200 rpm) at 30°C for 30 min. The soil suspension was then serially diluted up to 10–6 using sterile saline. Before plating, the samples were agitated at maximum speed using the vortex. An aliquot of 100 μl of each dilution was evenly spread over the surface of isolation media namely nutrient agar (NA), pseudomonas agar (PA), azotobacter media (AM) and azospirillum agar (AS) (HiMedia, India). Plates were incubated at 30°C for 12–24 h. The rhizobacterial colonies appeared on the plates were counted. Further, the isolates were subculture and purified to store at -80°C in 15% glycerol for a longer period. The gram staining of the bacterial isolates were performed and observed under light microscope (40X, Motic BA410 trinocular Microscope).

### Screening for *In vitro* Plant Growth Promoting (PGP) Traits

#### Phosphate Solubilisation

To determine the phosphate solubilising activity, rhizobacterial isolates were spotted onto Pikovskaya’s agar media (HiMedia, India). After 3 days of incubation at 28 ± 2°C, isolates that induced clear zone around the colonies were considered as positive (Katznelson and Bose, 1959). Quantification of tri-calcium phosphate solubilization was carried out using ammonium-molybdate reagent (Fiske and Subbarow, 1925) in liquid Pikovskaya’s medium. Absorbance values were obtained using the calibration curve with KH2PO4 at 650 nm for each isolates in 96-well microtiter plate by using multimode reader (Varioskan flash, Thermo Scientific, USA). The pH variation in the medium during the growth of each isolate was also observed.

#### Indole Acetic Acid (IAA) Production

The production of IAA was determined by both qualitative and quantitative assay as described by Gordon and Weber (1951). The bacterial isolates were grown in minimal salt (MS) medium amended with 100 μg ml^-1^ L-tryptophan for 72 h on the orbital shaker. The MS medium contained (per liter) 1.36 g KH2PO4, 2.13 g Na2HPO4, 0.2 g MgSO4.7H2O and trace elements. To measure the amount of IAA produced, bacterial supernatant was added to Salkowski’s reagent (35% HClO4 containing 10 mM FeCl3) in 1:2 ratio. After 25 min, the samples were read at 530 nm absorbance by using 96-well microtiter plate in multimode reader (Varioskan flash, Thermo Scientific, USA). Development of pink or red color indicates IAA production. Concentration of IAA produced by the bacterial isolates was compared to the standard curve of commercial IAA (Sigma–Aldrich, USA).

#### Siderophore Production

The siderophore of the bacterial isolates was determined by using chrome azurol S (CAS) agar plate assay. Briefly, inoculum (5 μl) of bacterial isolates were spotted onto the CAS agar plates and incubated at 28 ± 2°C for 72 h. Siderophore production was assessed on the basis of change in color of the medium from blue to orange (Schwyn and Neilands, 1987). Quantitative estimation of siderophores was performed by CAS-shuttle assay in which 0.5 ml of culture supernatant was mixed with 0.5 ml of CAS reagent (Payne, 1994). Absorbance was measured at 630 nm by using 96-well microtiter plate in multimode reader (Varioskan flash, Thermo Scientific, USA) against a reference consisting of 0.5 ml of uninoculated broth and 0.5 ml of CAS reagent. Siderophore content in the aliquot was calculated by using the following formula:

%siderophore⁢ units=Ar−As/Ar×100

Where, Ar = absorbance of the reference at 630 nm (CAS reagent) and As = absorbance of sample at 630 nm.

#### Ammonia Production

Ammonia production was determined both qualitatively and quantitatively as described by Cappuccino and Sherman (1992). For estimation, freshly grown bacterial isolates were inoculated in test tubes with 10 ml of peptone water and incubated for 48 h at 28 ± 2°C. After incubation, 1 ml of each culture was transferred to1.5 ml microtubes and 50 μl of Nessler’s reagent [10% HgI_2_; 7% KI; 50% aqueous solution of NaOH (32%)] was added in each microtube. The development of faint yellow color indicates a small amount of ammonia and deep yellow to brownish indicates maximum production of ammonia. The production of ammonia was measured at 450 nm using standard curve of ammonium sulfate in the range of 0.1–5 μmol ml^-1^by multimode reader (Varioskan flash, Thermo Scientific, USA).

#### Aminocyclopropane-1-carboxylate (ACC) Deaminase Activity

Aminocyclopropane-1-carboxylate deaminase acitivity was performed by qualitative assay using Dworkin and Foster (DF) salt medium (Dworkin and Foster, 1958) with 3 mM filter sterilized ACC as a sole nitrogen source (Sigma–Aldrich, USA). The 3 mM ACC solution was spread over the agar plates and bacterial isolates were spot inoculated on it. The bacterial growth on the plates was observed after 2 days incubation at 28°C (Naik et al., 2008).

#### Protease Activity

The protease activity was determined using skim milk agar medium which contains (per liter) 5 g pancreatic digest of casein, 2.5 g yeast extract, 1 g glucose, 7% skim milk solution and 15 g agar. Rhizobacterial isolates were spot inoculated and after 2 days incubation at 28°C, proteolytic activity was identified by clear zone around the cells (Smibert and Krieg, 1994).

#### Cellulase Activity

The rhizobacterial isolates were screened for cellulase production by plating onto M9 MS medium (HiMedia, India) agar amended with (per liter) 10 g cellulose and 1.2 g yeast extract. The bacterial colonies were observed after 8 days of incubation at 28°C. The clear halos formed by the colonies considered as positive for cellulase production (Cattelan et al., 1999).

### Molecular Identification of Potent PGPR

For molecular identification of the potent strains, PCR amplification of 16S rRNA gene was performed using universal primers PA (5′-AGAGTTTGATCCTGGCTCAG-3′) and 1492R (5′-GGTTACCTTGTTACGACTT-3′) (Weisburg et al., 1991). For PCR reaction, a single bacterial colony was picked up with a sterile toothpick and mixed in the PCR cocktail.PCR cocktail (40 μl) contained 1XTaq DNA polymerase buffer, 1 U of Taq DNA polymerase, 0.2 mM of each dNTP, 1.5 mM MgCl2, 0.2 μM of each primer and a single bacterial colony for template DNA. Amplifications were performed in thermocycler (Proflex PCR system, Applied Biosystems, USA) programmed with an initial denaturation at 94°C for 7 min, followed by 35 cycles of 30 s at 94°C, 30 s at 54°C and 1 min at 72°C with an extension of 72°C for 7 min. 5 μl aliquot of each amplification product was electrophoresed on a 1.7% agarose gel in 1XTAE buffer at 50 V for 45 min stained with ethidium bromide. PCR products were visualized under BioDoc-ItTM Imaging System (UVP, Cambridge, UK). The PCR product was purified by GenElute PCR clean up kit (Sigma–Aldrich, USA) and sent to Xcelris Genomics for sequencing located at Ahmedabad, India. The bacterial strains were identified by BLAST analysis. Calculation of the level of sequence similarity was performed using EzTaxon server 2.1 (Chun et al., 2007) and submitted to Genbank.

The sequences of 16S rRNA gene along with their closest homology sequences were aligned with the assist of multiple sequence alignment program CLUSTAL W (Higgins et al., 1992). The reference sequences were obtained from the Genbank database. Pairwise evolutionary distances were computed with the help of Kimura’s 2 parameter model (Kimura, 1980). The phylogenetic tree was constructed by neighbour-joining (NJ) method using MEGA 6 program with bootstrap values based on the 1000 replications of the original dataset (Felsenstein, 1985).

### Plant Growth Promoting Experiment

#### Plant Material

Six months old vegetatively propagated tea clones were obtained from TTRI, Tea Research Association, Assam, India and maintained in the greenhouse in polythene sleeves of size 15–17.7 cm layflat, 20–25 cm long and 150 gage thick. Three different types of tea clones, viz., TV1, TV19, and TV20 were used for the PGP experiments. TV1 is Assam-China hybrid standard clone, TV 19 is Cambod hybrid yield clone and TV 20 is Cambod hybrid standard clone (Das et al., 2012).

#### Preparation of Bacterial Inoculums and Treatment

To prepare bacterial inoculum, the bacterial isolate was cultured in a 500 ml flask containing 200 ml nutrient broth (HiMedia, India) and allowed to grow aerobically in shaking incubator at 180 rpm for 48 h at 30°C. The bacterial suspension was then diluted in sterilized distilled water to a final concentration of 10^8^–10^9^ cfu ml^-1^. The resulting suspensions were used to treat tea plants under greenhouse conditions. In greenhouse, the PGP experiment was performed in completely randomized design on three commercial tea clones TV1, TV19, and TV20. For each clone there were five treatments and an untreated control and each treatment contained three plant replicates. The bacterial inoculums (10^8^–10^9^ cfu ml^-1^) were applied in the form of soil drenching once in a month for three times. The soil of the tea sleeves were non-sterile. The plants were harvested after 6 months of inoculation. Shoot and root elongation, biomass production and number of tea leaves were compared with the uninoculated control plants.

### Data Analysis

The total isolates producing different PGP traits profile is represented as Venn diagram using the multiple dataset analysis feature of Vennture software (Martin et al., 2012). Plant growth promoting experiment of three different tea clones were conducted in a completely randomized design. The principal component analysis (PCA, is calculated based on eigen values and eigen vectors in a matrix) were performed on the datasets to evaluate the relationship between the samples to analyze the bacterial treatment for PGP. Moreover, fold change analysis was also performed in MetaboAnalyst 3.0 software (Xia et al., 2015) to compare the absolute value changes between two group means and output values are plotted in log2 scale, so that same fold change (up/down-regulated) will have the same distance to the zero baseline.

Further, the plant growth promoting datasets were subjected to two-way ANOVA using triplicate value to evaluate the significant difference in each plant growth promoting parameter between treated/inoculated and control (untreated/uninoculated plants) across the all three clones. The statistical analysis was performed with the SPSS software (SPSS 18.0, SPSS Inc., Chicago, IL, USA).

## Results

### Isolation of Rhizobacteria from Tea Rhizosphere Soil

The rhizobacteria were isolated from tea rhizosphere samples in different isolation media used. A total of 217 bacteria were isolated from six different tea estates located in Assam. The number of rhizobacteria isolated from each tea garden and their cfu range were summarized in the **Table 1**. Moreover, the soil texture of soil samples collected was identified according to the USDA (2010) soil classification with their pH.

**Table 1:** The location, soil type and number of bacteria isolated from tea rhizosphere soil samples collected from the tea estate of Assam.

Tea garden	Location	Soil type (USDA)	pH	CFU g^-1^ range	Number of isolates
Sonapur tea estate	26°06′56.40″N 91°58′33.18″E	Sandy loam	4.1	8 × 10^4^–1.2 × 10^6^	38
Khetri tea estate	26°06′53.81″N 92°05′27.74″E	Sandy loam and clay	4.6	7.6 × 10^4^–2 × 10^5^	33
Tocklai tea growing area	26°45′18.40″N 94°13′16.92″E	Sandy loam	4.1	8 × 10^4^–2.1 × 10^6^	35
Teok tea estate	26°36′29.41″N 94°25′42.59″E	Sandy loam	5.2	8 × 10^4^–3.5 × 10^6^	27
Hatikhuli tea estate	26°34′55.94″N 93°24′43.15″E	Clay loam and silty	4.3	11 × 10^4^–2.8 × 10^6^	63
Difaloo tea estate	26°36′29.41″N 93°35′03.96″E	Sandy loam	4.3	6 × 10^4^–2 × 10^6^	21

### Screening of Rhizobacteria for PGP Traits

**FIGURE 1:**
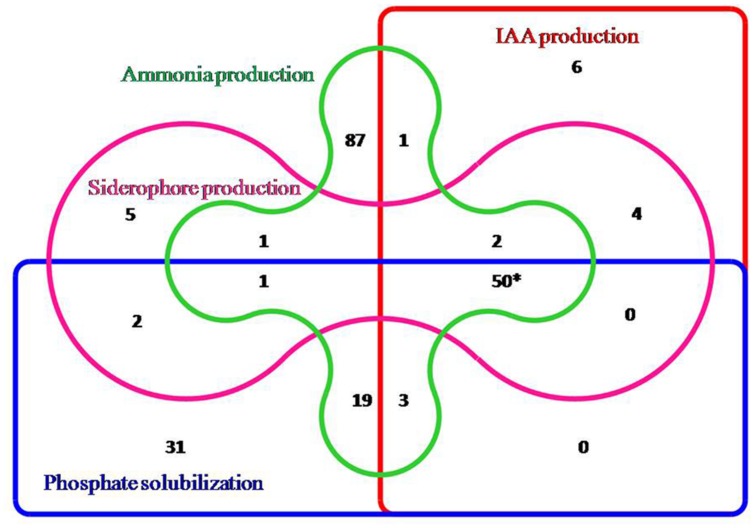
**Venn diagram representation of rhizobacterial isolates showing positive results for different PGP traits (^∗^50 isolates showing the positive results for all the PGP traits)**.

All 217 isolates were preliminary screened for different PGP traits out of which 106 isolates showed phosphate solubilizing ability, 65 isolates showed siderophore production, 66 isolates were positive for indole acetic acid (IAA) and 164 were positive for ammonia production. However, based on the preliminary screening, 50 isolates showed positive result for all the four traits, i.e., siderophore production, phosphate solubilisation, IAA production, and ammonia production and five isolates did not exhibit any PGP traits. Hence, out of 217 isolates 212 isolates exhibited atleast one PGP trait and the data was depicted by the Venn diagram representation (**Figure 1**). The 50 isolates which showed all the PGP traits were selected as potential rhizobacteria and further estimated for siderophore production, IAA production, phosphate solubilisation and ammonia production. All the PGP traits tested for 50 isolates are summarized in **Table 2**.

**Table 2:** Screening of selected rhizobacterial isolates for *in vitro* plant growth promoting traits.

Sl No	Strain code	Gram reaction	Cell shape	IAA production (μg ml^-1^)	Siderophore production^b^ (%)	Phosphate solubilization^c^ (μg ml^-1^)	Ammonia production^d^ (μmol ml^-1^)	ACC deaminase	Protease production^f^	Cellulase production
(1)	KH45	-	Rod	43.6 ± 0.1	38.6 ± 1.4	193.4 ± 1.8	3.3 ± 0.1	+	+	+
(2)	HK21	+	Cocci	18.3 ± 1.1	19.5 ± 0.3	115.6 ± 3.0	4.1 ± 0.04	+	+	+
(3)	SN30	+	Rod	16.5 ± 0.2	24.4 ± 0.6	160.6 ± 4.9	4.4 ± 0.1	^-^	+	^-^
(4)	TG30	-	Cocci	12.2 ± 0.2	14.6 ± 0.5	112.3 ± 4.1	4.1 ± 0.3	+	^-^	^-^
(5)	TG24	-	Cocci	12.3 ± 0.6	21.9 ± 1.7	133.8 ± 1.7	4.5 ± 0.1	^-^	+	+
(6)	TG27	-	Rod	14.6 ± 1.1	21.9 ± 0.9	143.8 ± 1.7	4.5 ± 0	+	^-^	^-^
(7)	HK25	-	Cocci	12.6 ± 1.1	17.0 ± 0.1	124.7 ± 3.9	4.5 ± 0	+	+	+
(8)	DT1	-	Cocci	19.6 ± 0.5	11.7 ± 0.2	182.2 ± 0.7	4.3 ± 0.1	^-^	^-^	^-^
(9)	TT6	-	Rod	65.3 ± 0.3	32.8 ± 1.3	249.5 ± 2.3	4.1 ± 0.1	+	+	+
(10)	KH34	-	Rod	11.0 ± 0.6	17.0 ± 0.1	65.5 ± 2.1	3.4 ± 0.6	^-^	^-^	^-^
(11)	HK54	-	Rod	14.2 ± 1.7	12.1 ± 1.8	73.7 ± 1.7	4.2 ± 0.1	^-^	^-^	^-^
(12)	DT15	-	Cocci	22.7 ± 0.5	23.4 ± 0.7	186.0 ± 1.9	3.7 ± 0.1	^-^	+	^-^
(13)	HK27	+	Cocci	12.2 ± 0.1	7.3 ± 0.6	168.5 ± 3.7	4.2 ± 0.2	^-^	^-^	^-^
(14)	KH49	+	Cocci	14.6 ± 0.1	21.9 ± 0.3	112.1 ± 1.1	4.9 ± 0.1	^-^	^-^	^-^
(15)	SN22	-	Rod	13.7 ± 0.1	12.1 ± 1.3	124.8 ± 2.3	0.8 ± 0.1	+	+	^-^
(16)	HK28	-	Cocci	14.3 ± 0.7	12.9 ± 1.0	129.7 ± 1.7	4.2 ± 0.04	+	^-^	^-^
(17)	HK31	-	Rod	12.3 ± 0.1	19.5 ± 0.9	77.3 ± 3.3	4.5 ± 0	+	+	^-^
(18)	SN27	+	Rod	24.8 ± 0	17.0 ± 0.5	146.4 ± 1.0	4.5 ± 0	+	^-^	^-^
(19)	SN18	+	Rod	10.2 ± 0.2	19.0 ± 1.3	165.1 ± 1.1	4.2 ± 0.2	+	+	^-^
(20)	HK8	-	Rod	13.0 ± 1.2	14.6 ± 0.3	135.2 ± 0.7	0.4 ± 0	^-^	^-^	^-^
(21)	SN25	+	Rod	12.2 ± 0.2	17.0 ± 1.0	150.0 ± 4.6	3.8 ± 0.1	+	+	+
(22)	HK18	-	Cocci	17.3 ± 0.6	17.0 ± 1.6	123.9 ± 3.3	4.5 ± 0	+	+	+
(23)	HK36	+	Rod	15.3 ± 0.5	14.6 ± 0.3	140.8 ± 6.9	4.5 ± 0	+	^-^	+
(24)	DT18	-	Rod	31.6 ± 0.5	14.5 ± 0.5	182.9 ± 2.7	4.5 ± 0	^-^	^-^	^-^
(25)	DT2	-	Rod	10.7 ± 0.7	18.1 ± 0.6	76.6 ± 2.7	4.5 ± 0	^-^	+	^-^
(26)	HK33	-	Cocci	12.2 ± 0.7	17.0 ± 0.2	108.5 ± 5.2	4.5 ± 0	+	^-^	^-^
(27)	HK51	-	Rod	11.3 ± 0.1	12.1 ± 0.1	134.2 ± 0.7	3.9 ± 0.2	^-^	^-^	+
(28)	SN29	+	Cocci	87.9 ± 0.8	39.8 ± 0.9	195.7 ± 2.3	4.4 ± 0.1	+	+	+
(29)	HK20	-	Rod	34.3 ± 1.2	17.0 ± 0.3	167.8 ± 4.3	4.5 ± 0	+	^-^	^-^
(30)	DT13	-	Rod	20.0 ± 0.8	11.7 ± 0.2	156.3 ± 3.6	4.3 ± 0.1	^-^	^-^	^-^
(31)	HK26	-	Cocci	15.3 ± 0.3	17.1 ± 0.8	148.7 ± 2.3	4.5 ± 0	+	^-^	^-^
(32)	HK23	-	Cocci	16.3 ± 0.1	33.8 ± 1.5	120.2 ± 1.5	0.9 ± 0	+	+	+
(33)	SN28	-	Cocci	27.6 ± 2.3	21.9 ± 0.5	168.7 ± 3.8	4.2 ± 0.2	+	+	+
(34)	SN23	+	Cocci	12.3 ± 0.3	19.5 ± 0.3	168.9 ± 0.4	4.4 ± 0.1	^-^	+	+
(35)	HK38	+	Rod	11.5 ± 1.1	12.1 ± 1.3	130.5 ± 3.2	4.1 ± 0.1	^-^	+	+
(36)	HK37	+	Cocci	10.7 ± 1.2	7.3 ± 2.0	95.4 ± 0.7	2.9 ± 0.1	+	^-^	^-^
(37)	HK17	-	Cocci	12.2 ± 0.4	24.3 ± 0.4	176.1 ± 5.8	4.2 ± 0.2	^-^	+	+
(38)	HK9	-	Cocci	13.7 ± 0.5	21.9 ± 0.3	187.8 ± 3.8	4.5 ± 0	+	+	+
(39)	SN26	-	Rod	19.2 ± 1.1	19.4 ± 0.3	146.1 ± 4.9	4.4 ± 0.1	+	^-^	^-^
(40)	HK19	-	Rod	11.0 ± 0.5	17.0 ± 1.0	97.4 ± 2.3	4.4 ± 0.1	^-^	+	+
(41)	SN24	-	Rod	13.7 ± 1.4	21.9 ± 0.5	134.3 ± 3.0	3.7 ± 0.1	^-^	^-^	^-^
(42)	HK30	-	Rod	16.4 ± 0.5	19.5 ± 0.4	175.3 ± 2.8	4.3 ± 0.2	+	+	+
(43)	DT9	+	Cocci	10.2 ± 0.5	12.1 ± 0.1	147.9 ± 1.9	1.6 ± 0.1	^-^	^-^	^-^
(44)	HK32	+	Rod	12.3 ± 0.1	9.6 ± 0.6	113.0 ± 3.0	4.5 ± 0	+	^-^	+
(45)	HK2	-	Cocci	27.4 ± 0.2	26.8 ± 1.4	155.2 ± 1.5	4.1 ± 0.04	+	+	+
(46)	KH18	-	Cocci	14.7 ± 0.1	12.1 ± 1.8	162.8 ± 4.1	4.5 ± 0	^-^	^-^	^-^
(47)	HK29	-	Rod	22.5 ± 0.5	19.5 ± 0.5	156.0 ± 1.1	4.2 ± 0.2	+	+	^-^
(48)	TT19	+	Rod	15.3 ± 0.5	24.2 ± 0.2	174.9 ± 2.0	0.5 ± 0.04	+	^-^	^-^
(49)	TG1	-	Rod	92.5 ± 0.2	36.9 ± 0.2	298.2 ± 1.8	4.4 ± 0.1	+	+	+
(50)	DT23	-	Rod	11.0 ± 1.2	16.9 ± 0.3	125.1 ± 2.8	4.4 ± 0.2	^-^	^-^	^-^

*Values are mean of three replicates SD. means showed no activity and means showed activity present.*

### Estimation of Phosphate Solubilisation

**FIGURE 2:**
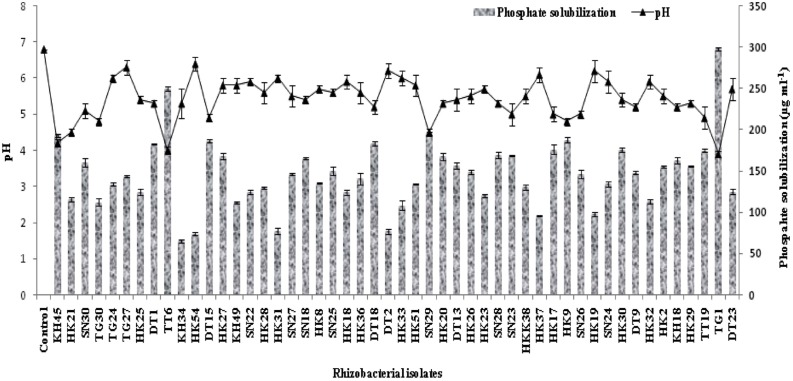
**Quantitative estimation of 50 selected phosphate solubilising rhizobacteria with their pH profile after 5 days of incubation**.

For phosphate solubilisation, the isolates were observed up to 5 days from incubation and there was significant decrease in the pH level of the media. Most of the isolates showed good phosphate solubilising ability but 22 isolates were able to solubilize 150 μg ml^-1^ or more calcium phosphate in the medium (**Figure 2**). Isolates KH45, TT6, TG1, and SN29 showed significantly higher phosphate solubilisation than the other isolates.

### Estimation of IAA, Siderophore and Ammonia Production

**FIGURE 3:**
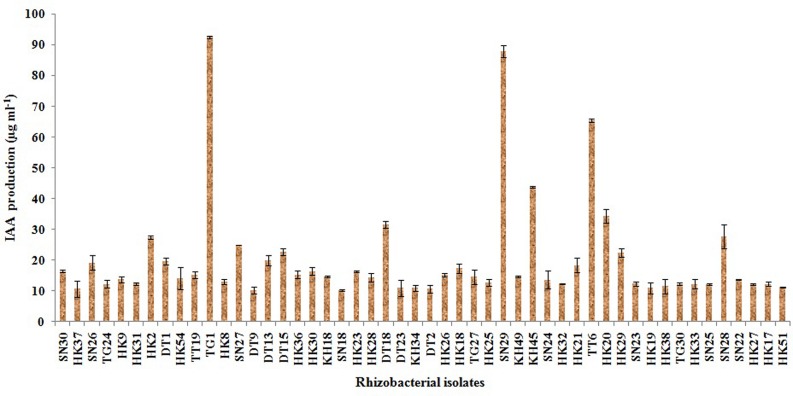
**Quantitative estimation of IAA production by 50 selected rhizobacterial isolates**.

**FIGURE 4:**
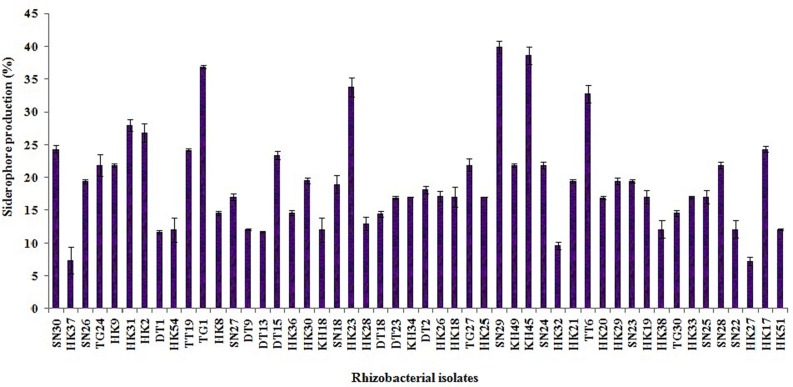
**Quantitative estimation of siderophore production by 50 selected rhizobacterial isolates**.

**FIGURE 5:**
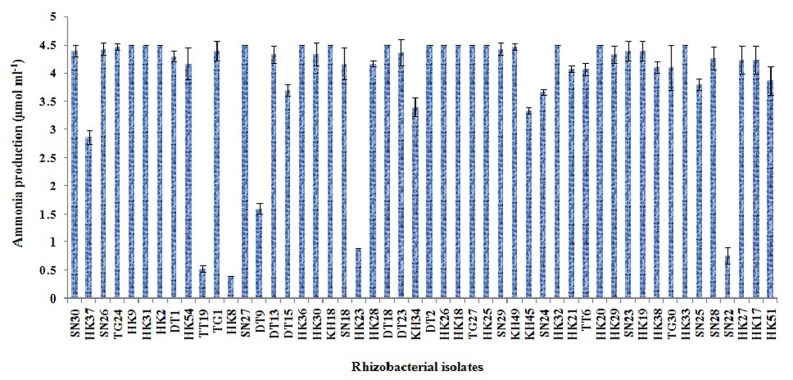
**Quantitative estimation of ammonia production by 50 selected rhizobacterial isolates**.

Out of the 50 isolates, 12 isolates produced 20 μg ml^-1^ or more IAA after 72 h of incubation with 100 μg ml^-1^ supplement of L-tryptophan. Siderophore production was estimated for all the selected 50 isolates. Only five isolates were able to produce above 30% of siderophore units. Most of the isolates showed ammonia production in the range of 4–4.5 μmol ml^-1^. Isolate KH49 showed the maximum ammonia production with 4.9 μmol ml^-1^ among all the isolates. All the isolates producing IAA, siderophore, and ammonia are represented in **Figures 3**–**5**.

### Screening of ACC Deaminase, Protease, and Cellulase Assay

All the 50 isolates were further screened qualitatively for ACC deaminase, protease and cellulase production, out of which 29 (58%) isolates showed positive results for ACC deaminase activity, 25 (50%) isolates showed protease production and 21 (42%) isolates showed cellulase production. The results are shown in **Table 2**.

### Selection of Potent PGPR for Greenhouse Plant Growth Promoting Experiment

Four isolates from *in vitro* PGP screening, KH45, TT6, SN29, and TG1 showed higher amount of IAA production among all the isolates out of which TG1 produced maximum IAA which was 92.5 μg ml^-1^. These four isolates also showed significantly higher phosphate solubilisation as depicted in **Table 2**. Five isolates producing more than 30% units of the siderophore were KH45, TT6, SN29, HK23, and TG1. Though the isolate HK23 produced 33.8% siderophore unit but it produced less amount of IAA and lower levels of phosphate solubilisation compared to the other four isolates which were recorded for the best production of siderophore. Moreover, HK23 could not show positive result for all the PGP traits tested. Isolate KH49 showed the best ammonia production with 4.9 μmol ml^-1^. Isolate KH49 also produced less amount of IAA, siderophore and phosphate solubilisation compared to the isolates KH45, TT6, SN29, and TG1. Moreover, the isolate KH49 did not show positive result for all the *in vitro* traits tested. On the basis of these PGP screening, the isolates KH45, TT6, SN29, and TG1 were considered as most potent isolates as all the four showed very prominent results in all the PGP traits tested for this study. The efficacy of these four isolates was further evaluated by greenhouse plant growth promoting experiment on tea plants.

### Molecular Identification of the Potent PGPR

**FIGURE 6:**
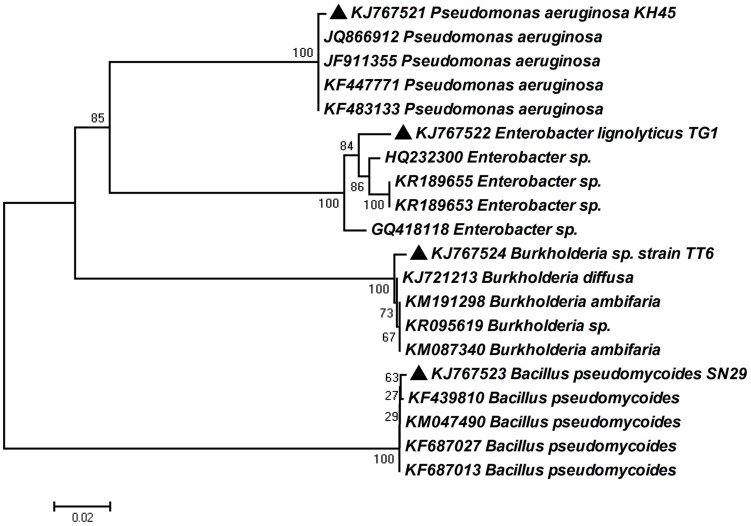
**Phylogenetic relationship based on partial 16S rRNA gene sequence of four bacterial strains selected for plant growth promotion (PGP) with their best matches in the EZ-Taxon database.** The phylogenetic tree was constructed using neighbor-joining method. A bootstrap analysis was performed with 1000 replicates and the scale bar indicates the number of differences in base composition among sequences.

In order to determine the identity of four most potent PGP isolates, 16s rRNA gene was amplified by colony PCR method and sequences were obtained from Xcelris Genomics sequence service provider. The sequences were then compared using BLAST tool. Based on BLAST analysis of the 16S rRNA gene homology, the isolates were identified as *Pseudomonas aeruginosa* strain KH45, *Enterobacter lignolyticus* strain TG1, *Bacillus pseudomycoides* strain SN29 and *Burkholderia* sp. strain TT6. The sequences were deposited in the Genbank database under the accession numbers KJ767521, KJ767522, KJ767523, and KJ767524 (**Table 3**). A neighbor-joining dendogram was generated using the sequences from the four rhizobacteria and the representative sequences from the databases (**Figure 6**).

**Table 3:** Molecular identification of 16S rRNA gene, accession numbers and their origin of four selected potent rhizobacterial strains for greenhouse plant growth promotion (PGP).

Strains code	Genbank accession no	Closest sequence similarity	*E*-value	Origin
KH45	KJ767521	*Psedumonas aeruginosa* (99%)	0	Khetri tea estate, Assam, India
TG1	KJ767522	*Enterobacter lignolyticus* (99%)	0	Tocklai tea growing area, Assam, India
SN29	KJ767523	*Bacillus pseudomycoides* (99%)	0	Sonapur tea estate, Assam, India
TT6	KJ767524	*Burkholderia* sp. (99%)	0	Teok Tata tea estate, Assam, India

### Plant Growth Promoting Experiment

**FIGURE 7:**
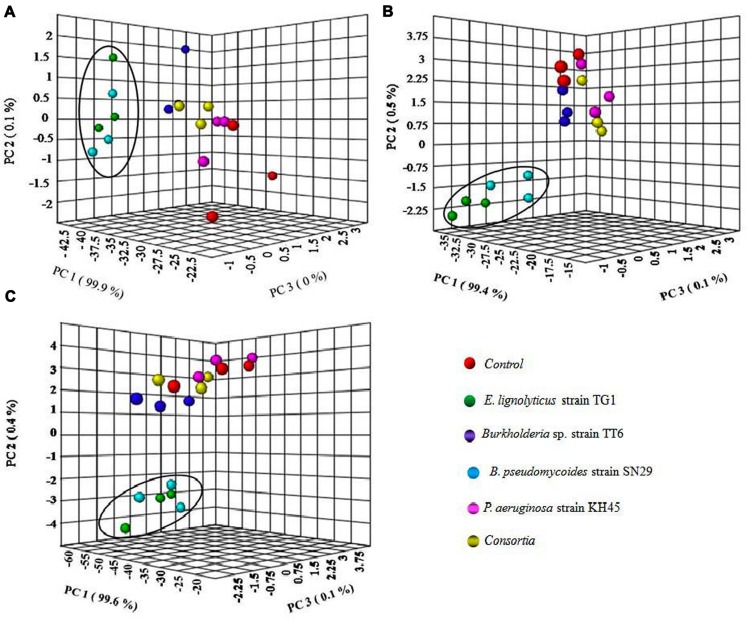
**Clustering relationship of inoculated with strains TG1, TT6, SN29, KH45 and consortia with uninoculated control plants based on the PCA analysis of greenhouse tea plant growth promoting experiment for (A) TV1 clone, (B) TV19 clone, and (C) TV20 clone**.

The PCA analysis of the five different treatments for greenhouse PGP showed that the two groups of treatments TG1 and SN29 clustered together and other three groups of treatments TT6, KH45, and consortia appeared with the control in all the three TV1, TV19, and TV20 clones (**Figures 7A–C**).

To validate the PCA results, fold change analysis was performed to analyze the level of increase in the plant growth parameters of bacterial inoculated plants compared to the uninoculated control plants. In all the three tea clones TV1, TV19, and TV20, the bacterial inoculum of *E. lignolyticus* strain TG1 showed significant increase in number of fold change compared to the other bacterial inoculums. TG1 showed maximum root biomass production with an increase in 4.3-fold, shoot biomass with increase in 3.1-fold, root length by 2.2-fold and shoot length by 1.6-fold in TV19 clone. However, TG1 showed maximum number of leaves in TV20 clone by increase in 1.7-fold compared to the control plants. The graph of fold change analysis and their log values are provided as a Supplementary File 1: Figures S1A–O and Tables S2A–O.

**FIGURE 8:**
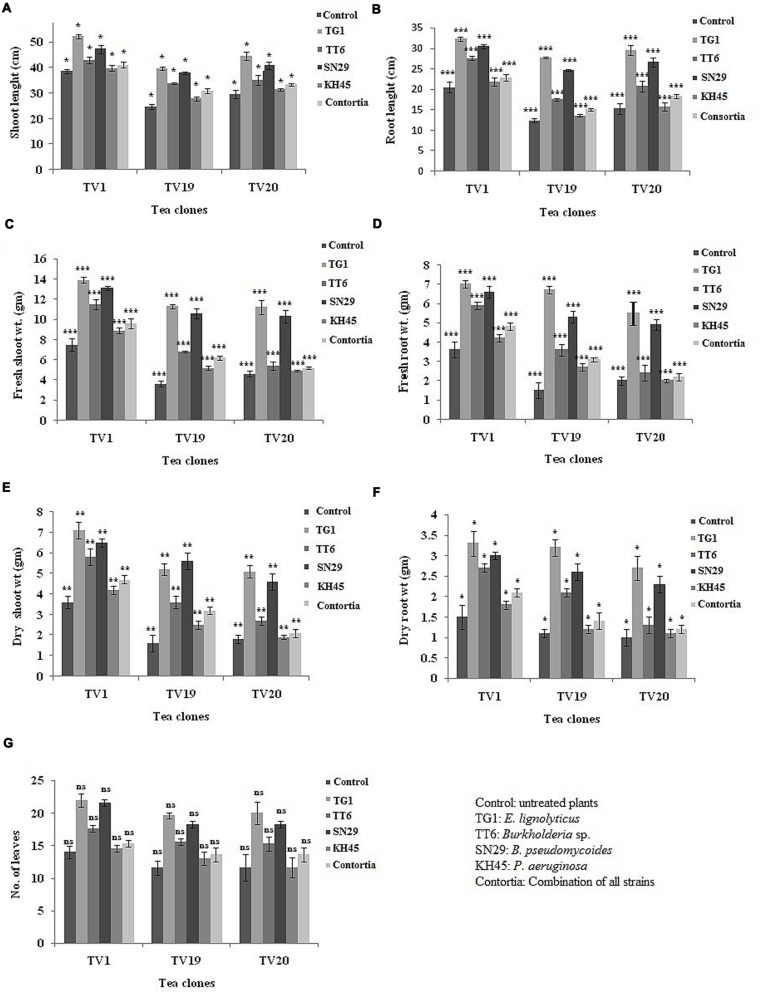
**Evaluation of greenhouse plant growth promoting experiment to show the effect of treatments in three tea clones TV1, TV19, and TV20 for (A) shoot length, (B) root length, (C) fresh shoot weight, (D) fresh root weight, (E) dry shoot weight, (F) dry root weight and (G) number of leaves.** (^∗^*P*-value 0.05–0.01, ^∗∗^*P*-value 0.01–0.001, ^∗∗∗^*P*-value less than 0.001 and ^ns^*P*-value greater than 0.05; see Supplementary Table S3).

Further, two-way ANOVA analysis was carried out to analyze the effect of treatment and clone on the growth parameters. In this study, three clones, i.e., TV19 as yield clone and TV1 and TV20 as standard clones were evaluated in greenhouse PGP experiment. All the plants inoculated with the isolates showed significant difference at *P* < 0.05 in all the growth parameters. The tea plants of TV19 clone inoculated with *E. lignolyticus* strain TG1 showed maximum increase in the shoot and root length and biomass production (**Figures 8A–G**; see Supplementary File 2: Figures S2A–E and Supplementary File 3: Table S3). There was no significant difference in number of leaves comparing among all the three tea clones.

## Discussion

In the present investigation, 217 rhizobacterial isolates from the tea rhizosphere soil were isolated and screened for different *in vitro* PGP traits out of which 50 isolates showed excellent *in vitro* PGP activity and five isolates did not exhibit any PGP traits analyzed. Further, based on the quantitative analysis of 50 isolates for wide array of PGP traits, four most promising rhizobacterial strains belongs to different genera were selected for further study. The 16S rRNA gene analysis of these four bacteria TG1, TT6, SN29, and KH45 has revealed 99% sequence similarity with *E. lignolyticus*, *Burkholderia* sp., *B. pseudomycoides* and *P. aeruginosa*, respectively. These bacterial isolates showing various *in vitro* PGP attributes multiple beneficial mechanisms of action and plant growth promoting efficacy was further evaluated in greenhouse experiment. These selected isolates showed excellent *in vitro* PGP activity like phosphate solubilisation, IAA production, siderophore production, and ammonia production. One of these beneficial characters is IAA synthesized by PGPR which is responsible for the increased adventitious root number and length and also the root volume by which roots can provide a large amount of nutrients to the plant and its higher root exudates in turn benefits the bacteria (Ramos et al., 2008). In our study, *E. lignolyticus* strain TG1 was recorded as the best strain for IAA production (92.5 μg ml^-1^) that exceeds the IAA levels of previously reported works (Yasmin et al., 2007; Vasconcellos et al., 2010; Ribeiro and Cardoso, 2012). The PGPR, *E. cloacae* in rice (Mehnaz et al., 2001), *Enterobacter* sp. in sugarcane (Mirza et al., 2001) showed IAA production. Moreover, this strain solubilises inorganic phosphate up to 298.2 μg ml^-1^ with significant decrease in pH. The solubilised inorganic phosphate is a promising attribute for the selection of bacteria capable of increasing available phosphorus in the rhizosphere. Liu et al. (2014) demonstrated that the most common and effective acids involved in the inorganic phosphate solubilisation are gluconic acid, lactic acid, malic acid, succinic acid, formic acid, citric acid, malonic acid, and tartaric acid. In our study, the solubilisation of calcium was observed due to significant decrease in pH of the culture medium which was probably responsible for production of organic acids by the bacteria. Phosphate solubilisation and siderophore production by different free-living rhizospheric bacteria, *Bacillus*, *Azotobacter*, and *Pseudomonas* was documented by Ahmad et al. (2008). Siderophore production is another important attribute for PGP. Rhizobacteria synthesize and release siderophore which are low molecular weight molecules that bind Fe^3+^ that can chelate Fe^3+^ and make it less available for other species in the microbial community of the rhizosphere. PGPR have been demonstrated to enhance the PGP very efficiently by producing extracellular siderophores which can control several plant diseases by depriving the pathogen of iron nutrition thus resulting in increased crop yield (O’Sullivan and O’Gara, 1992). The strain *B. pseudomycoides* showed the highest siderophore production (39.8%) among the four selected PGPR. Yu et al. (2011) reported, siderophore producing *B. subtilis* CAS15 strain showed growth promotion by stably colonized on pepper roots. Ammonia production also plays an important role in plant growth by accumulation of nitrogen and helps in increase of root and shoots growth and biomass production (Marques et al., 2010). ACC deaminase production of PGPR elicits the growth promotion by decreasing the level of ethylene production. Though ethylene is an essential metabolite for plant growth but in different stress condition the ethylene level is increased drastically which could negatively influence the plant growth. ACC is an immediate precursor molecule for ethylene production and the bacteria producing ACC deaminase could hydrolyse ACC into ammonia and α-ketobutyrate instead of ethylene (Ahemad and Kibret, 2014). Cellulase and protease production by PGPR could also be possible plant growth promoting factors. Cellulose is found most abundantly in plant biomass which can be decomposed by cellulase enzyme produced by microbes (Lynd et al., 2002). Moreover, protease producing microbes can also act as a biocontrol agent on protein cell wall bearing pathogens. These cellulase and protease producing microbes play a significant role in decomposition of organic matter, nutrient mineralization, and PGP (Lima et al., 1998). The bearing of these traits implies that these microbes have potential to use as biofertilizer for crop yield.

All the four isolates selected in this study for greenhouse experiment on the tea plants showed significant increase in all the growth parameters evaluated. The individual inoculum of the bacterial isolates showed most promising effect on the plants rather than the consortia except strain KH45. The multivariate PCA analysis of the greenhouse parameters showed relation between the treatment and growth parameters of the plants. The PCA analysis of the plants inoculated with the *E. lignolyticus* strain TG1 and *B. pseudomycoides* strain SN29 showed significantly higher PGP compared to the other isolates. To validate these results, the fold change analysis was performed to analyze the level of fold change of growth parameters of inoculated plants compared to the uninoculated control plants. The fold change analysis revealed that the *E. lignolyticus* strain TG1 exhibited highest increase in fold change in root and shoot length and biomass production of TV19 clone compared to the control. The TG1 showed 4.3-fold increase in root biomass production and 2.2-fold increase in root length which could be correlated with the *in vitro* production of IAA. Rhizobacterial IAA is an important metabolite which helps in root development and we have found that the isolate TG1 showed maximum *in vitro* IAA production. The rhizobacterial IAA also increases root exudation by loosening the plant root cell walls which inturn helps in the rhizobacterial colonization and growth (Glick, 2012). These results implied that the isolate TG1 helps in tea root development and it could be used as a potential biofertilizer agent in tea crop fields. Further, the two-way ANOVA analysis also justified the PGP results analyzed by PCA and fold change analysis. The effect of treatments on the PGP in different tea clones exhibited significant (*P* < 0.05) increase in growth promotion of tea plants except the number of leaves and the isolate TG1 showed the best growth promotion among all the isolates tested.

The genus *Enterobacter* was previously reported for PGP. *E. arachidis* was reported as a novel species of genus *Enterobacter* from rhizosphere soil of groundnut showing PGP activity (Madhaiyan et al., 2010). *E. asburiae* was isolated from mustard rhizosphere soil which was reported as fungicide tolerant and endowed with PGP traits (Ahemad and Khan, 2010). Madhaiyan et al. (2013) reported the complete genome sequence of *Enterobacter* sp. strain R4-368, an endophytic N-fixing γ-proteobacterium isolated from surface sterilized roots of *Jatropha curcas* L. showing strong growth-promoting activity by increasing plant biomass and seed yields. Also, *E. radicincitans* DSM16656 isolated from the phyllosphere of winter wheat has been shown to promote the growth of several plant species (Witzel et al., 2012). Although, there were several reports in genus *Enterobacter* as PGPR but there has been no evidence reported for the *E. lignolyticus* as plant growth promoting agent in the tea crop field.

## Conclusion

In the present study, effective isolates of rhizobacteria from tea rhizosphere soil was selected which could be a useful component of sustainable agricultural management. Although, all four rhizobacteria have been demonstrated for their PGP potential in tea plant, isolate *E. lignolyticus* strain TG1 was found to have superiority over other isolates in terms of growth promotion. Hence, these indigenous rhizosphere associated soil microbial inhabitants with wide array of PGP activity could be beneficial for tea plantation of Northeast India. However, further experiments are needed to determine the effectiveness of these rhizobacterial isolates under different field conditions to study the nature of interaction with other soil native microflora and the host plant.

## Author Contributions

DT supervised the research work and guided the experimental design. PJH provided the research work suggestion. JD performed the laboratory and field experiments, analysed the data. DT and JD prepared the manuscript.

## Conflict of Interest Statement

The authors declare that the research was conducted in the absence of any commercial or financial relationships that could be construed as a potential conflict of interest.
