# Supplementary material for: Assessment of Culturable Tea Rhizobacteria Isolated from Tea Estates of Assam, India for Growth Promotion in Commercial Tea Cultivars
Source: Front Microbiol. 2015 Nov 10;6:1252. doi: 10.3389/fmicb.2015.01252 (PMC4639606; doi:10.3389/fmicb.2015.01252)
Supplement: Supplementary file 1 [file Table_1.PDF]

Supplementary 3

Table S3 | Effect of bacterial inoculation on *in vivo* plant growth promotion in three different tea clones TV1, TV19 and TV20.

Treatment	Tea Clones	Shoot length (cm)^{a*}	Root length (cm)^{b***}	Shoot fresh wt. (gm)^{c***}	Root fresh wt. (gm)^{d***}	Shoot dry wt. (gm)^{e**}	Root dry wt. (gm)^{f*}	No. of leaves^{gns}
Control		38.5±0.9	20.5±1.3	7.5±0.6	3.7±0.4	3.7±0.4	1.5±0.4	14.0±1.0
E. lignolyticus		52.2±0.8	32.3±0.6	13.9±0.4	7.0±0.2	7.1±0.4	3.4±0.3	22.0±1.0
Burkholderia sp.		42.9±1.2	27.6±0.5	11.5±0.5	5.9±0.3	5.9±0.4	2.8±0.1	17.7±0.6
B. pseudomycoides	TV1	47.2±1.7	30.6±0.5	13.2±0.3	6.7±0.4	6.5±0.2	3.0±0.2	21.7±0.6
P. aeruginosa		39.7±1.1	21.8±1.0	8.9±0.4	4.2±0.3	4.2±0.3	1.8±0.4	14.7±0.6
Consortia		41.0±1.2	22.9±0.8	9.7±0.6	4.8±0.2	4.8±0.3	2.1±0.2	15.3±0.6
Control		24.7±1.2	12.5±0.5	3.6±0.4	1.6±0.4	1.6±0.4	1.1±0.1	11.7±1.2
E. lignolyticus		39.7±0.8	27.8±0.2	11.3±0.3	6.7±0.3	5.3±0.3	3.2±0.3	19.7±0.6
Burkholderia sp.		33.8±0.5	17.6±0.4	6.9±0.2	3.6±0.4	3.7±0.4	2.1±0.1	15.7±0.6
B. pseudomycoides	TV19	37.9±0.6	24.8±0.2	10.6±0.5	5.3±0.4	5.6±0.4	2.6±0.2	18.3±0.6
P. aeruginosa		27.8±0.8	13.6±0.3	5.2±0.3	2.8±0.2	2.5±0.3	1.2±0.2	13.0±1.0
Consortia		30.8±1.1	15.1±0.4	6.2±0.3	3.1±0.1	3.3±0.3	1.4±0.2	13.7±1.2
Control		29.7±1.5	15.3±1.4	4.7±0.4	2.0±0.2	1.8±0.2	1.0±0.2	11.7±2.1
E. lignolyticus		44.5±1.5	29.5±1.3	11.3±0.7	5.5±0.6	5.1±0.4	2.7±0.3	20.0±1.7
Burkholderia sp.		35.1±1.9	20.8±1.3	5.4±0.4	2.4±0.4	2.8±0.3	1.4±0.3	15.3±1.2
B. pseudomycoides	TV20	40.8±1.4	26.7±1.2	10.3±0.7	4.9±0.4	4.7±0.5	2.4±0.3	18.3±0.6
P. aeruginosa		31.4±0.5	15.8±1.0	5.0±0.2	2.0±0.1	1.9±0.1	1.1±0.1	11.7±1.5
Consortia		33.4±0.5	18.4±0.5	5.2±0.2	2.2±0.2	2.2±0.2	1.2±0.1	13.7±1.2
P value		*P*= 0.025	*P*= 0.0001	*P*= 0.0001	*P*= 0.0001	*P*= 0.002	*P*= 0.045	*P*= 0.889

a, b, c, d, e, f, g Values are mean of three replicates ± SD
Two-way ANOVA was conducted with P values; *(P value 0.05-0.01), **(P value 0.01-0.001), ***(P value less than 0.001) and ^{ns}(P value greater than 0.05)
