# Supplementary material for: Assessment of Culturable Tea Rhizobacteria Isolated from Tea Estates of Assam, India for Growth Promotion in Commercial Tea Cultivars
Source: Front Microbiol. 2015 Nov 10;6:1252. doi: 10.3389/fmicb.2015.01252 (PMC4639606; doi:10.3389/fmicb.2015.01252)
Supplement: Supplementary file 2 [file Data_Sheet_1.PDF]

Supplementary file : 1

Clone TV1

Fold change analysis of treatment TG1

Table S2 (a)

	Fold Change	log2(FC)
Dry root wt	2.2444	1.1664
Dry shoot wt	1.9455	0.96011
Fresh root wt	1.9091	0.93289
Fresh shoot wt	1.8533	0.89012
Root length	1.5756	0.65591
No. of leaves	1.5714	0.65208
Shoot length	1.355	0.43827

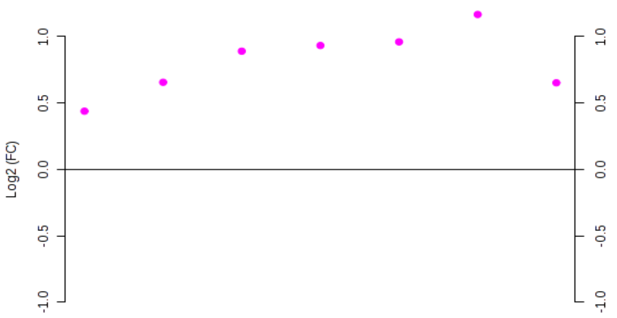

Figure S1 (a)

Compounds

Fold change analysis of treatment TT6

Table S2 (b)

	Fold Change	log2(FC)
Dry root wt	1.8444	0.88319
Fresh root wt	1.6182	0.69437
Dry shoot wt	1.6	0.67807
Fresh shoot wt	1.5333	0.61667
Root length	1.3463	0.42904
No. of leaves	1.2619	0.3356
Shoot length	1.1134	0.155

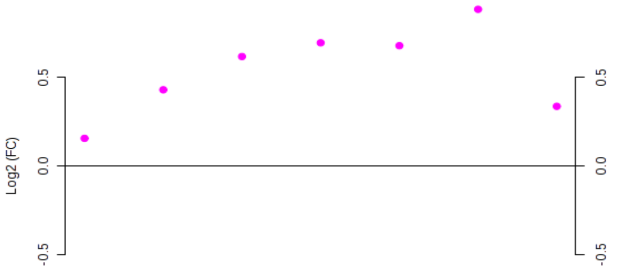

Figure S1 (b)

Compounds

Fold change analysis of treatment SN29

Table S2 (c)

	Fold Change	log2(FC)
Dry root wt	2.0222	1.0159
Fresh root wt	1.8182	0.8625
Dry shoot wt	1.7727	0.82597
Fresh shoot wt	1.7556	0.81193
No. of leaves	1.5476	0.63005
Root length	1.4911	0.57634
Shoot length	1.2268	0.29495

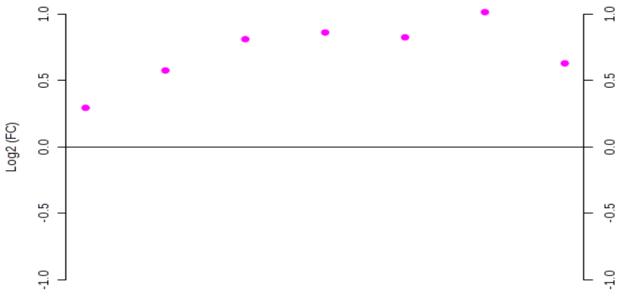

Figure S1 (c)

Compounds

Fold change analysis of treatment KH45

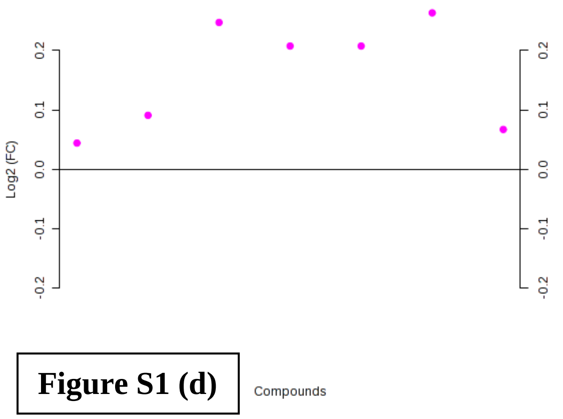

Figure S1 (d)

Table S2 (d)

	Fold Change	log2(FC)
Dry root wt	1.2	0.26303
Fresh shoot wt	1.1867	0.24691
Fresh root wt	1.1545	0.20732
Dry shoot wt	1.1545	0.20732
Root length	1.065	0.090908
No. of leaves	1.0476	0.067114
Shoot length	1.0312	0.044281

Fold change analysis of treatment Consortia

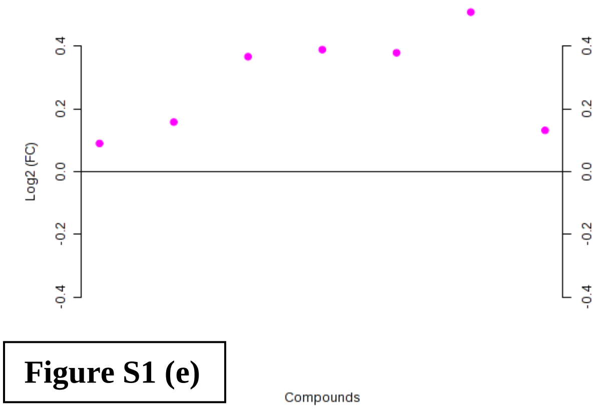

Figure S1 (e)

Table S2 (e)

	Fold Change	log2(FC)
Dry root wt	1.4222	0.50815
Fresh root wt	1.3091	0.38857
Dry shoot wt	1.3	0.37851
Fresh shoot wt	1.2889	0.36613
Root length	1.1154	0.15762
No. of leaves	1.0952	0.13124
Shoot length	1.0641	0.089592

Clone TV20

Fold change analysis of treatment TG1

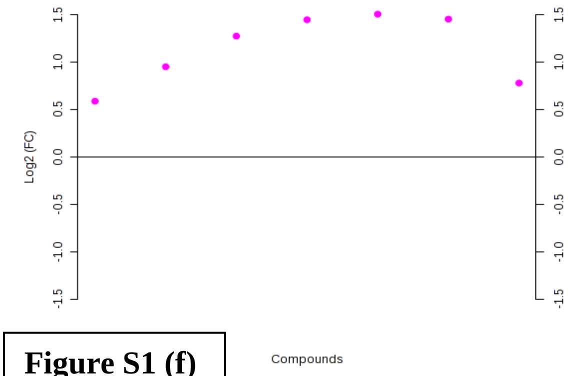

Figure S1 (f)

Table S2 (f)

	Fold Change	log2(FC)
Dry shoot wt	2.8333	1.5025
Dry root wt	2.7333	1.4507
Fresh root wt	2.7213	1.4443
Fresh shoot wt	2.4143	1.2716
Root length	1.9303	0.94881
No. of leaves	1.7143	0.77761
Shoot length	1.5011	0.58604

Fold change analysis of treatment TT6

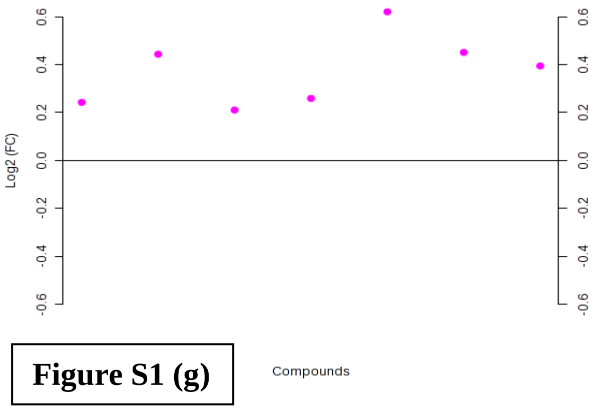

Figure S1 (g)

Table S2 (g)

	Fold Change	log2(FC)
Dry shoot wt	1.537	0.62015
Dry root wt	1.3667	0.45066
Root length	1.3595	0.44305
No. of leaves	1.3143	0.39428
Fresh root wt	1.1967	0.25909
Shoot length	1.1831	0.24263
Fresh shoot wt	1.1571	0.21057

Fold change analysis of treatment SN29

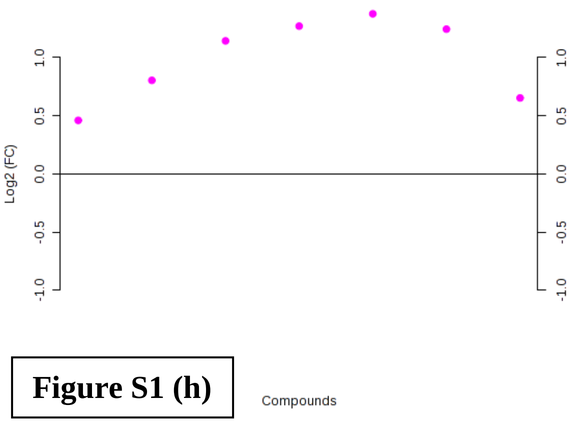

Figure S1 (h)

Table S2 (h)

	Fold Change	log2(FC)
Dry shoot wt	2.5926	1.3744
Fresh root wt	2.4098	1.2689
Dry root wt	2.3667	1.2429
Fresh shoot wt	2.2071	1.1422
Root length	1.7451	0.80331
No. of leaves	1.5714	0.65208
Shoot length	1.3753	0.45973

Fold change analysis of treatment KH45

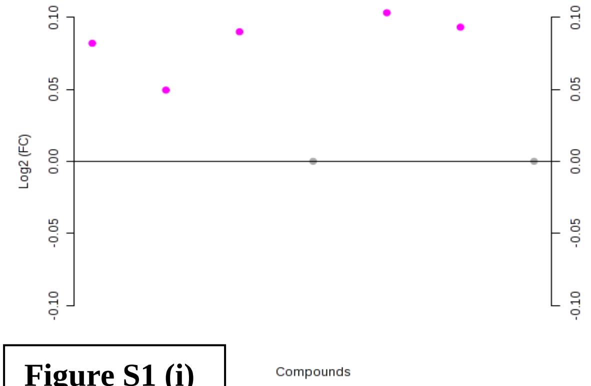

Figure S1 (i)

Table S2 (i)

	Fold Change	log2(FC)
Dry shoot wt	1.0741	0.10309
Dry root wt	1.0667	0.093109
Fresh shoot wt	1.0643	0.089886
Shoot length	1.0584	0.081922
Root length	1.0349	0.049433

Fold change analysis of treatment Consortia

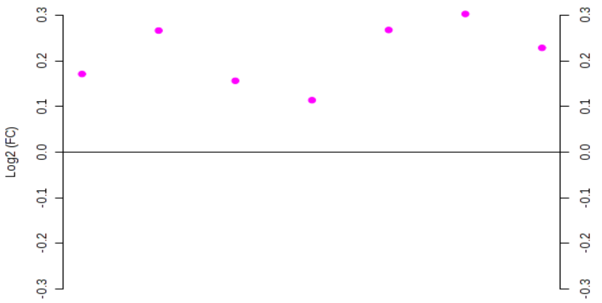

Figure S1 (j)

Table S2 (j)

	Fold Change	log2(FC)
Dry root wt	1.2333	0.30256
Dry shoot wt	1.2037	0.26748
Root length	1.2026	0.26617
No. of leaves	1.1714	0.22827
Shoot length	1.1258	0.17101
Fresh shoot wt	1.1143	0.15612
Fresh root wt	1.082	0.11366

Clone TV19

Fold change analysis of treatment TG1

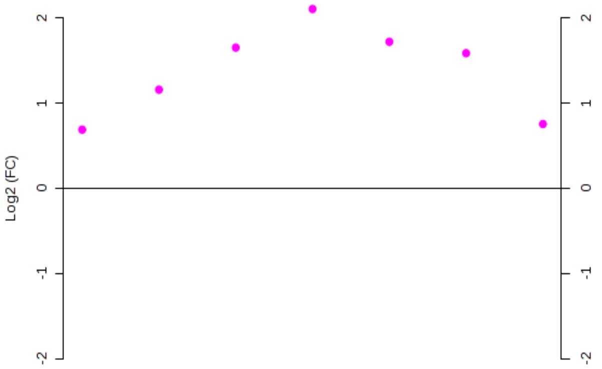

Figure S1 (k)

Table S2 (k)

	Fold Change	log2(FC)
Fresh root wt	4.2979	2.1036
Dry shoot wt	3.2917	1.7188
Fresh shoot wt	3.1389	1.6503
Dry root wt	3	1.585
Root length	2.2299	1.157
No. of leaves	1.6857	0.75336
Shoot length	1.6108	0.68779

Fold change analysis of treatment TT6

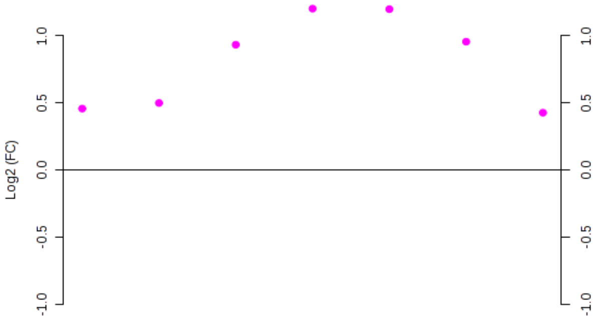

Figure S1 (l)

Table S2 (l)

	Fold Change	log2(FC)
Fresh root wt	2.2979	1.2003
Dry shoot wt	2.2917	1.1964
Dry root wt	1.9375	0.9542
Fresh shoot wt	1.9074	0.93161
Root length	1.4118	0.4975
Shoot length	1.3716	0.45588
No. of leaves	1.3429	0.42531

Fold change analysis of treatment SN29

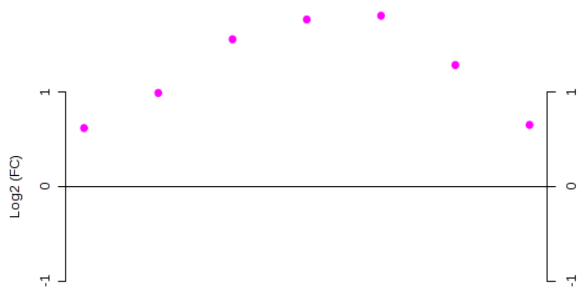

Figure S1 (m)

Table S2 (m)

	Fold Change	log2(FC)
Dry shoot wt	3.5	1.8074
Fresh root wt	3.4043	1.7673
Fresh shoot wt	2.9444	1.558
Dry root wt	2.4375	1.2854
Root length	1.9866	0.99032
No. of leaves	1.5714	0.65208
Shoot length	1.5351	0.61837

Fold change analysis of treatment KH45

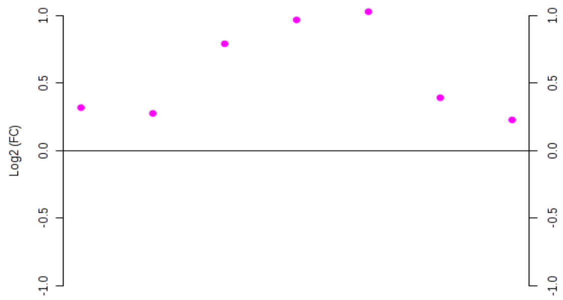

Figure S1 (n)

Table S2 (n)

	Fold Change	log2(FC)
Fresh root wt	1.766	0.82045
Dry shoot wt	1.5625	0.64386
Fresh shoot wt	1.4537	0.53973
Dry root wt	1.1562	0.20945
Shoot length	1.1284	0.17425
No. of leaves	1.1143	0.15612
Root length	1.0936	0.12906

Fold change analysis of treatment Consortia

Table S2 (o)

	Fold Change	log2(FC)
Dry shoot wt	2.0417	1.0297
Fresh root wt	1.9574	0.96897
Fresh shoot wt	1.7315	0.79201
Dry root wt	1.3125	0.39232
Shoot length	1.2473	0.31881
Root length	1.2112	0.27647
No. of leaves	1.1714	0.22827

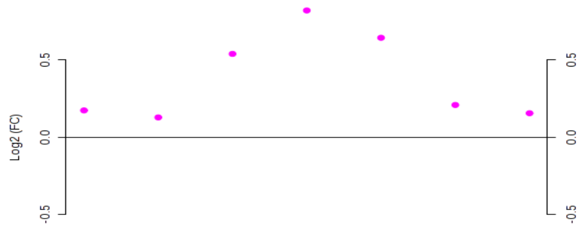

Figure S1 (o)

Note: In all figures the red circles represent features above the threshold and Important features selected by fold change analysis with threshold 1. The values are on log scale, so that both up-regulated and down regulated features can be plotted in a symmetrical way
