# Supplementary material for: Assessment of Culturable Tea Rhizobacteria Isolated from Tea Estates of Assam, India for Growth Promotion in Commercial Tea Cultivars
Source: Front Microbiol. 2015 Nov 10;6:1252. doi: 10.3389/fmicb.2015.01252 (PMC4639606; doi:10.3389/fmicb.2015.01252)
Supplement: Supplementary file 3 [file Image_1.PDF]

Supplementary file: 2

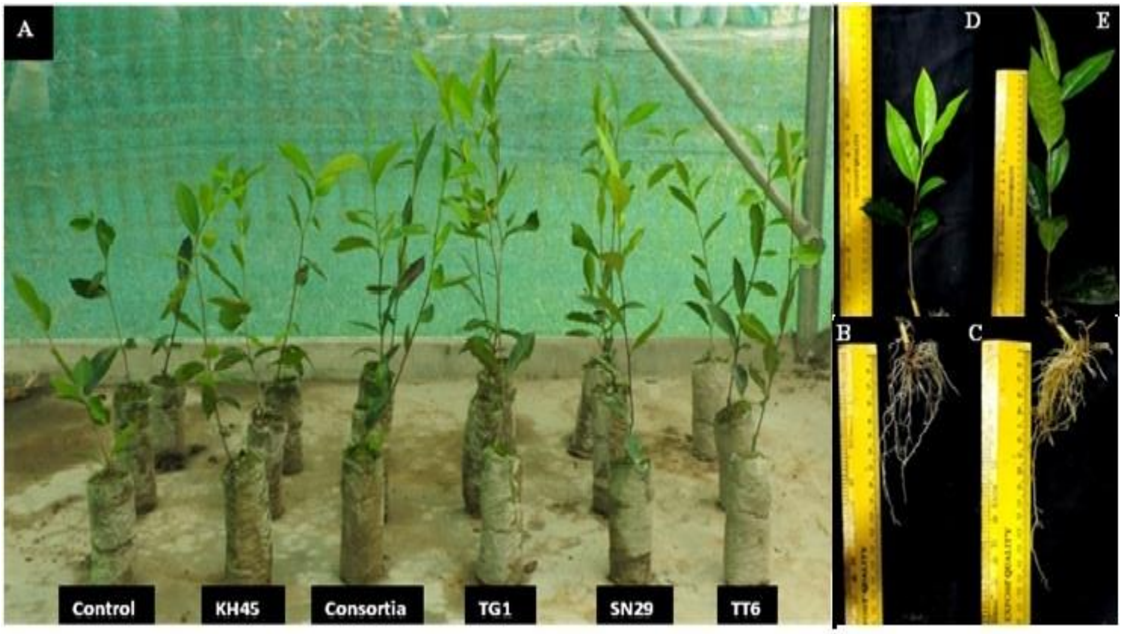

Figure S2 | Evaluation of different treatment of PGPR in *in-vivo* greenhouse experiment on tea plant (Clone TV1) (A) untreated (control plant), treated with *P. aeruginosa* strain KH45, consortia, *E. lignolyticus* strain TG1, *B. pseudomycoides* strain SN29, *Burkholderia* sp. strain TT6, (B) Root length of the control plant, (C) Root length of the TG1 treated plant, (D) Shoot length and leaves of the control plant and (E) Shoot length and leaves of treated plant.
